# Supplementary material for: Deep learning reveals a damage signalling hierarchy that coordinates different cell behaviours driving wound re-epithelialisation
Source: Development. 2024 Sep 24;151(18):dev202943. doi: 10.1242/dev.202943 (PMC11449448; doi:10.1242/dev.202943)
Supplement: Supplementary information [file develop-151-202943-s1.pdf]

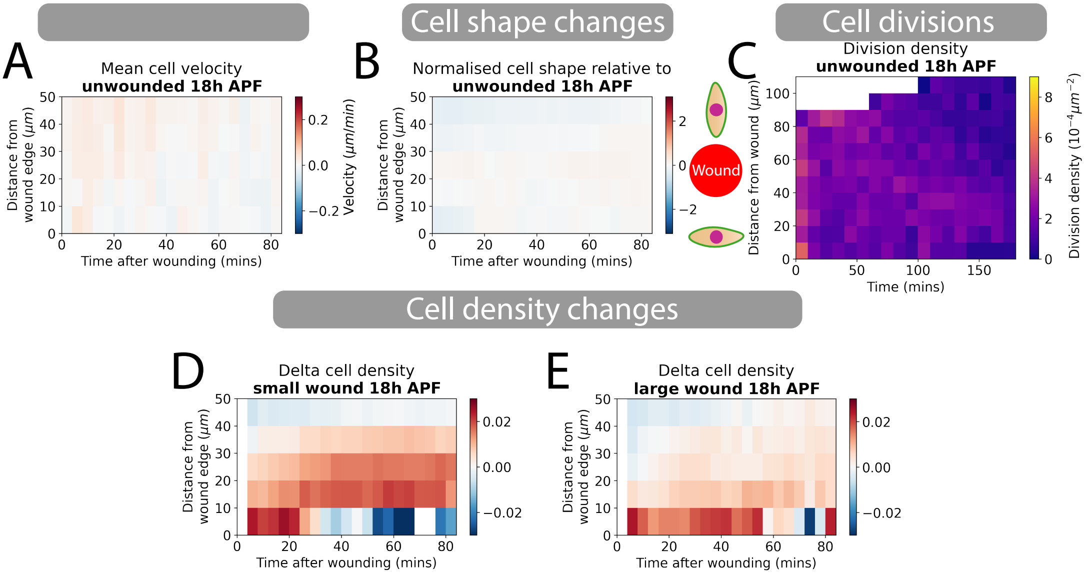

Fig. S1. Cell behaviour changes relative to "virtual wound" in unwounded tissues and changes in cell density in wild type small and large wounds.
A) Spatio-temporal heatmaps of cell velocity relative to wound centre for "virtual wounds", respectively. Blue regions indicate cells migrating away from the "wound", and red cells migrating towards the "wound". B) Spatio-temporal heatmaps of cell shape elongation relative to wounds for "virtual wounds", respectively. Blue regions indicate cells elongated perpendicular to "wounds", and red cells oriented towards the "wound". C) Heatmaps of the division density for "virtual wounds". D-E) Spatio-temporal heatmaps of difference in cell density relative to tissue average. Blue regions indicate cells are less dense than the rest of the tissue, and red cells are denser. All heatmaps are weighted means of a cell behaviour for each wound video with the weight corresponding to the visible area of tissue in videos. n=14 unwounded/"virtual wounds", n=8 small wounds and n=9 large wounds.

Development • Supplementary information

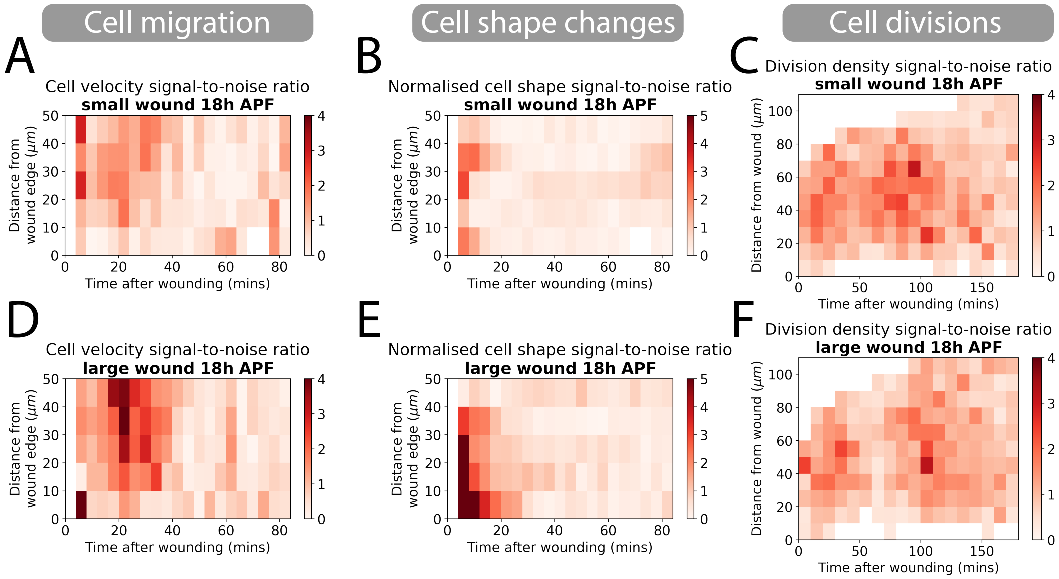

Fig. S2. Signal to noise ratio of cell behaviour around wild type wounds. A,D) Signal to noise ratio heatmaps of cell velocity relative to wound centre for small and large wounds, respectively. B,E) Signal to noise ratio heatmaps of cell shape elongation relative to wounds for small and large wounds, respectively. C,F) Signal to noise ratio heatmaps of the division density for small and large wounds.

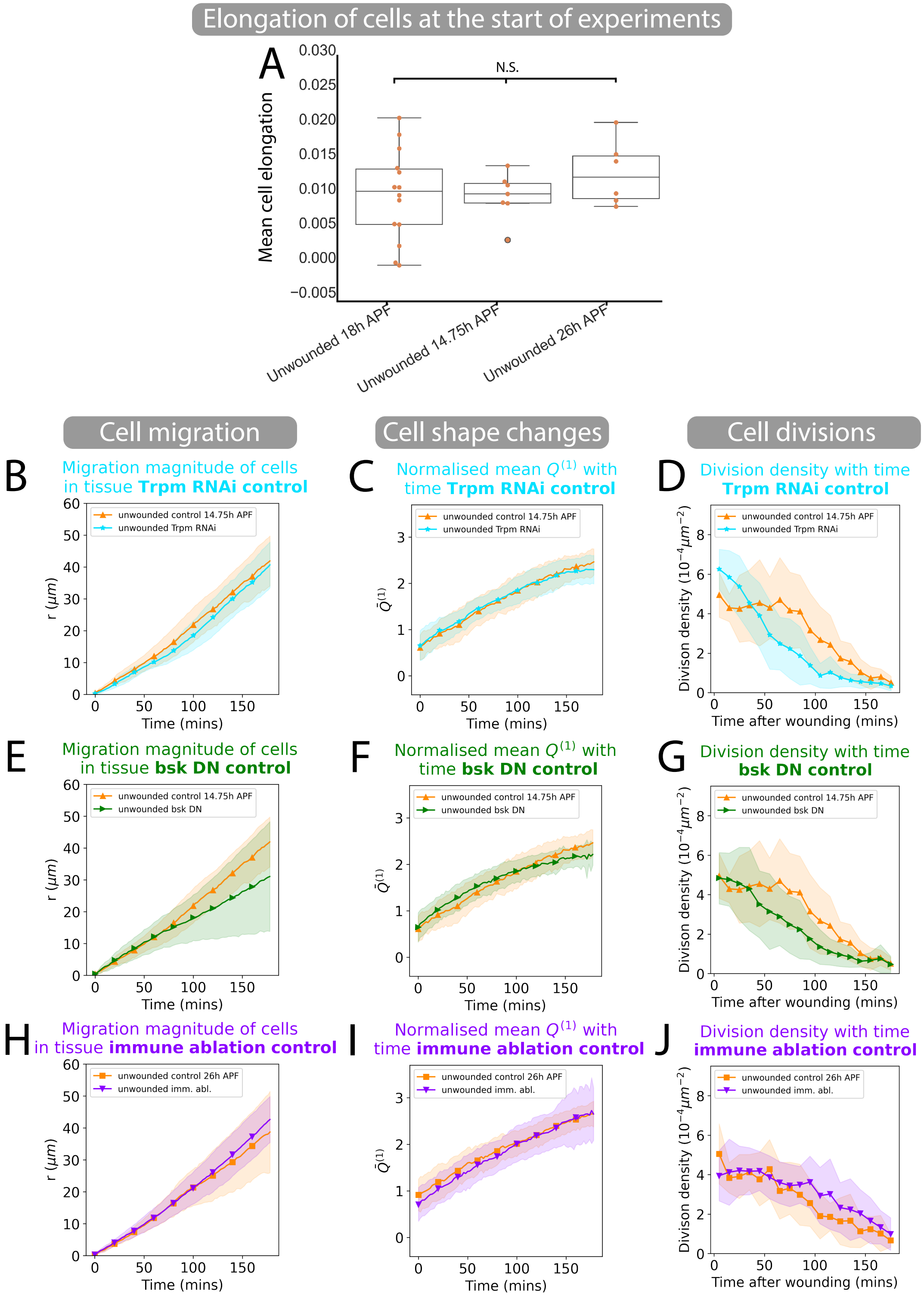

Development • Supplementary information

Fig. S3. Development of pupal wings at the start of imaging to confirm consistent staging across genotypes and quantify differences in cell behaviours.
A) Box plot of the distributions of initial mean cell elongation of pupal wing tissue relative to the P/D axis of the wing. Multiple t-test were performed but without the commonly used p value corrections as we are more concerned about type II errors. B,E,H) Mean migration of cells with time for control and mutant developing tissue. C,F,I) Mean q-tensor of cells with time for control and mutant developing tissue. D,G,J) density of cell divisions with time for control and mutant developing tissue (n=14 18h APF unwounded , n=7 14.75h APF unwounded , n=6 26h APF unwounded, n=10 TrpmRNAi unwounded, n=13 BskDN unwounded and n=15 immune cell ablated unwounded)

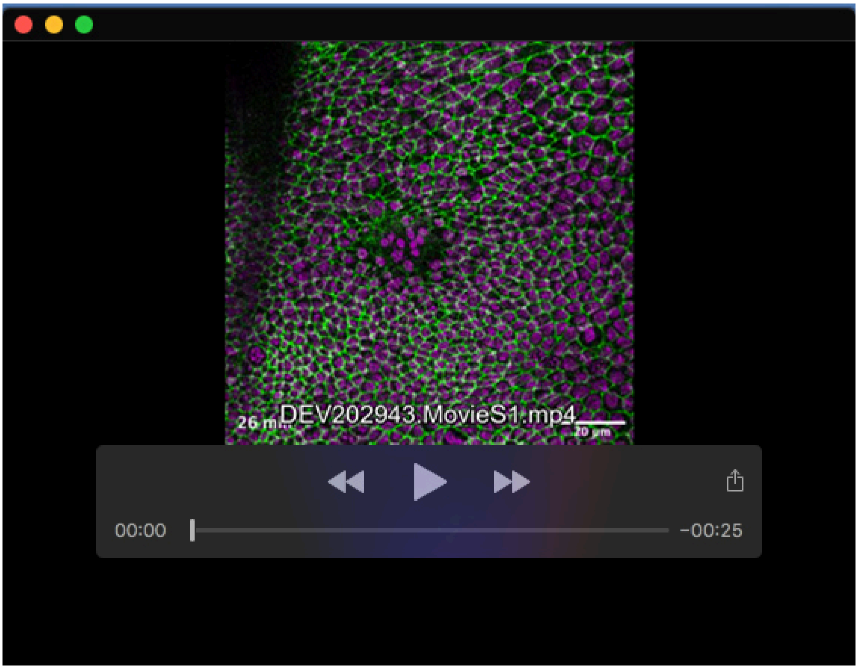

Movie 1. Time-lapse imaging of a small control wound in the pupal epithelium over 3 hours. - Projected from a 3D stack using the stack focus algorithm with a radius of 5 pixels. Green indicates E-cadherin-GFP and magenta indicates Histone2-RFP. One frame taken every 2 minutes. Scale bar: 20µm.

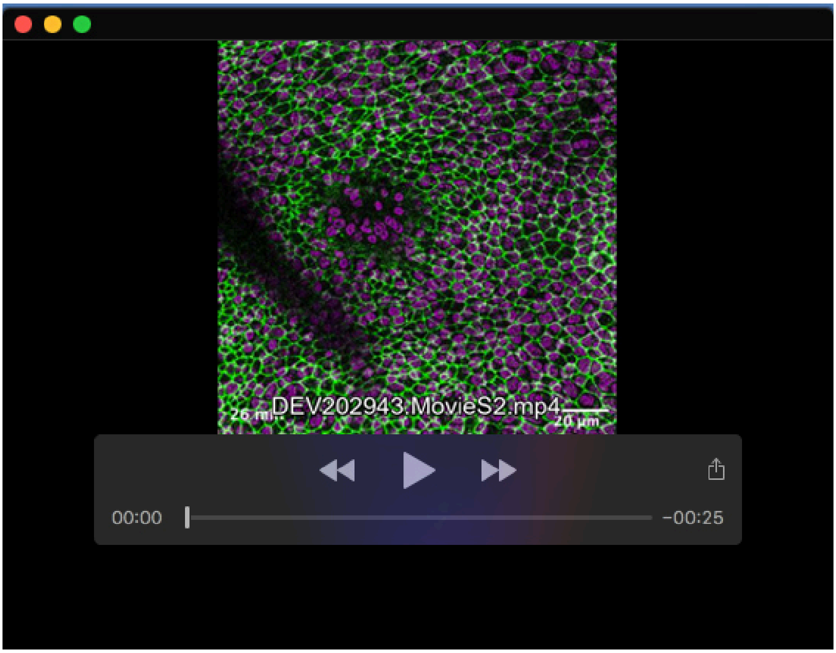

Movie 2. Time-lapse imaging of a large control wound in the pupal epithelium over 3 hours. - Projected from a 3D stack using the stack focus algorithm with a radius of 5 pixels. Green indicates E-cadherin-GFP and magenta indicates Histone2-RFP. One frame taken every 2 minutes. Scale bar: 20µm.

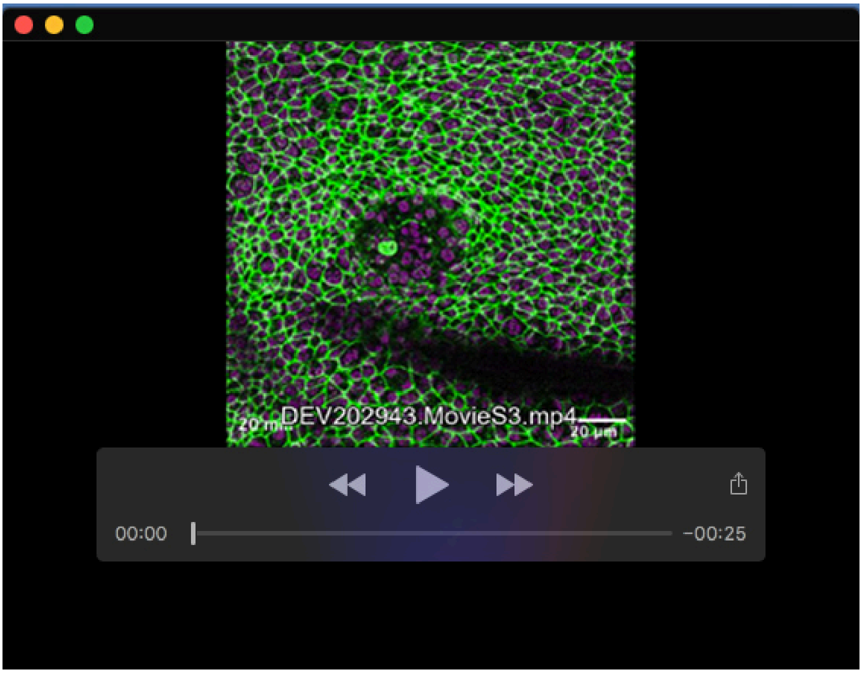

Movie 3. Time-lapse imaging of a large TrpmRNAi wound in the pupal epithelium over 3 hours. - Projected from a 3D stack using the stack focus algorithm with a radius of 5 pixels. Green indicates E-cadherin-GFP and magenta indicates Histone2-RFP. One frame taken every 2 minutes. Scale bar: 20µm.

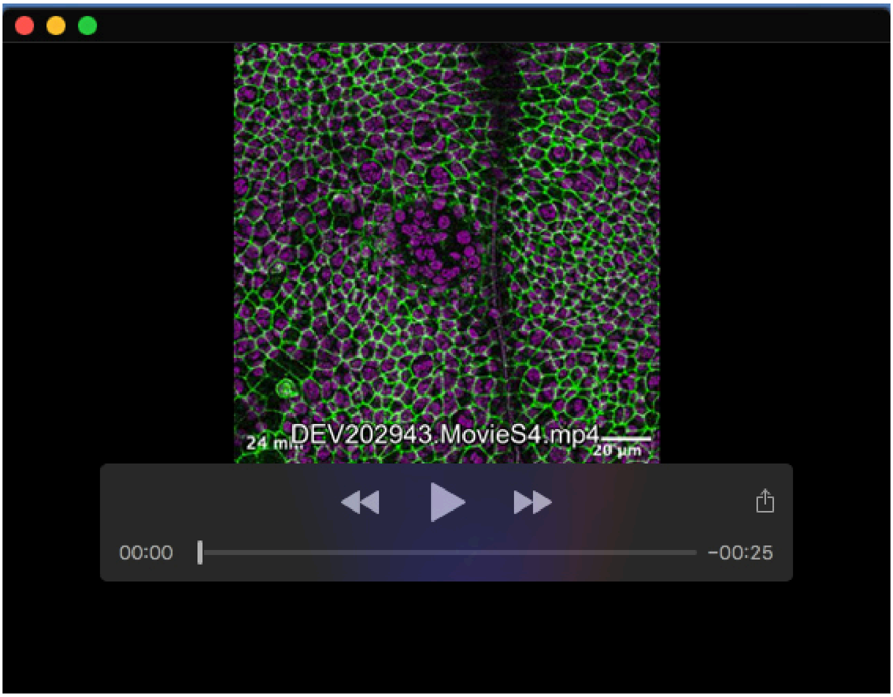

Movie 4. Time-lapse imaging of a large JNK knockdown wound in the pupal epithelium over 3 hours. - Projected from a 3D stack using the stack focus algorithm with a radius of 5 pixels. Green indicates E-cadherin-GFP and magenta indicates Histone2-RFP. One frame taken every 2 minutes. Scale bar: 20µm.

Development • Supplementary information

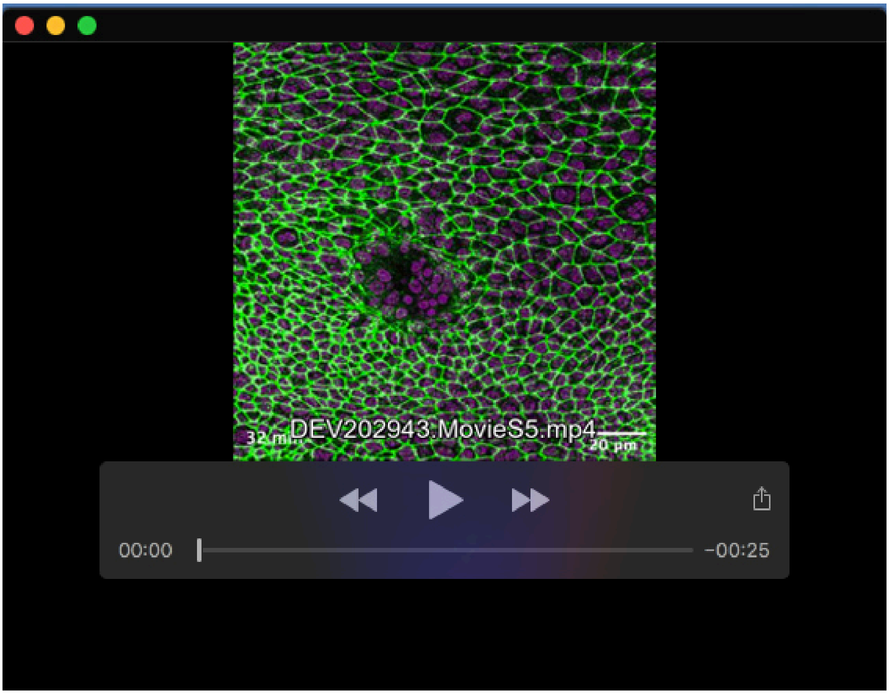

Movie 5. Time-lapse imaging of a large hemocyte ablated wound in the pupal epithelium over 3 hours. - Projected from a 3D stack using the stack focus algorithm with a radius of 5 pixels. Green indicates E-cadherin-GFP and magenta indicates Histone2-RFP. One frame taken every 2 minutes. Scale bar: 20µm.
