# Supplementary material for: Deep learning reveals a damage signalling hierarchy that coordinates different cell behaviours driving wound re-epithelialisation
Source: Development. 2024 Sep 24;151(18):dev202943. doi: 10.1242/dev.202943 (PMC11449448; doi:10.1242/dev.202943)
Supplement: Supplementary file 1 [file develop-151-202943_review_history.pdf]

Deep learning reveals a damage signalling hierarchy that coordinates different cell behaviours driving wound re-epithelialisation

Jake Turley, Francesca Robertson, Isaac V. Chenchiah, Tanniemola B. Liverpool, Helen Weavers and Paul Martin
DOI: 10.1242/dev.202943

Editor: Thomas Lecuit

Review timeline

Original submission:	10 April 2024
Editorial decision:	28 May 2024
First revision received:	10 July 2024
Accepted:	16 August 2024

Original submission

First decision letter

MS ID#: DEVELOP/2024/202943

MS TITLE: AI reveals a damage signalling hierarchy that coordinates different cell behaviours driving wound re-epithelialisation

AUTHORS: Jake Turley, Francesca Robertson, Isaac V. Chenchiah, Tanniemola B. Liverpool, Helen Weavers, and Paul Martin

I have now received all the referees reports on the above manuscript, and have reached a decision. The referees' comments are appended below, or you can access them online: please go to BenchPress and click on the 'Manuscripts with Decisions' queue in the Author Area.

The overall evaluation is positive and we would like to publish a revised manuscript in Development, provided that the referees' comments can be satisfactorily addressed. Please attend to all of the reviewers' comments in your revised manuscript and detail them in your point-by-point response. If you do not agree with any of their criticisms or suggestions explain clearly why this is so. If it would be helpful, you are welcome to contact us to discuss your revision in greater detail. Please send us a point-by-point response indicating your plans for addressing the referees' comments, and we will look over this and provide further guidance.

Reviewer 1

Advance summary and potential significance to field

In this study, Turley and colleagues applied various deep-learning based methodologies to perform a systematic and quantitative phenomenology of wound healing in the fly pupal wing. Using tools described in a previous publication (https://elifesciences.org/reviewed-preprints/87949), including U-net based improved segmentation and automatic detection of cell division, they performed an exhaustive characterisation in space and time of key drivers of wound healing (cell shape, cell migration, cell division) in a quite large number of movies of healing pupal wing in WT, upon perturbation of calcium signaling, JNK signaling and elimination of haemocytes. While the manuscript may not provide clear mechanistic/molecular understanding of which cellular effectors are affected by these pathways, it is a very interesting proof of concept of the usage of automatise

© 2024. Published by The Company of Biologists under the terms of the Creative Commons Attribution License (https://creativecommons.org/licenses/by/4.0/).

methodology to performed a very thorough and detailed characterisation of phenotype, including subtle differences in term of spatio-temporal distribution of cell behaviour, which to my knowledge was probably never performed to this extend in the context of wound healing.

The main novel features is the clear delineation of the effet of JNK signaling of cell shape and cell division (without strong impact on cell movement), the effect of haemocyte on cell migration, and the general impact of calcium on all the process. Despite the absence of detailed mechanisms explaining these quantitative phenotypes, I believe this article could be well positioned for Developmental readership and it exemplifies and sets interesting new standard for quantitative charaterisation of phenotype using AI. Somehow, this was already done in the context of morphogenesis through systematic segmentation and shear decomposition in the context of tissue elongation in the pupal wing, notum and embryo, but I believe this manuscript shows that one can start to extend these exhaustive approaches to a larger range of phenotype and higher number of movies when combined with AI. My main critic at this point is that some of the extracted features are relative hard to interpret in term of "phenotype" since they are themself emerging for a large range of cellular behaviours and I am wondering if other metrics (provided they are accessible with the dataset) may not help to go even deeper in the understanding of the phenotype. I realise that some of the these parameters may not be accessible without very intensive work (specially if these parameters would require flawless segmentation and tracking) and touch then the limit what one can currently do with reasonable manual curation and deep learning (and as such should be openly discussed here, see my suggestions below).

Comments for the author

Main suggestions :

1. So far, the manuscript has been mostly documenting cell division, cell speed, and cell shape. While each of these features are very relevant, I believe cell speed is relatively hard to interpret as it could emerge from many features, for instance the purse-string tension at the wound that could pull cells, the active crawling of cells (specially at the leading edge of the wound) and / or the movements driven by global cell shape changes and/or T1 transition/cell intercalation. As a result, it is relatively hard to interpret what are the key cellular effectors affected by the different perturbations. To be really exhaustive, one should ideally perform the "shear" decomposition (https://elifesciences.org/articles/07090) in radial coordinates around the wound in all the context described (which would give a "unambiguous" evaluation of the contribution of different cell behaviour to the closure), but admittedly this would be very tedious in all these movies and cellular contexts as it would require flaw-less segmentation for perfect accurate tracking (if I understood well, the authors did not do that, they just analyse cell shape at different point without considering cell identity other time, which can deal properly with ~5% segmentation error ? This was not crystal clear in the method).
As such, I would not necessarily recommend to do this analysis (unless the authors do have fully curated segmentation), but at least this limitation should be clearly discussed, specially if the point of the article is to show what can be done using these automatised methodologies.

2. Along this line, I realise one key feature not accessible right now is tissue fluidity and the rate of T1. Since this was already shown to play an essential role in wound healing (https://www.ncbi.nlm.nih.gov/pmc/articles/PMC6837871/) it would have been relevant to extract this feature. But this again requires segmentation and tracking (and may not be easily accessible at this stage).

To summarise point 1 and 2, I am suggesting to clarify what is exactly accessible in the dataset, and if the segmentation/tracking is indeed one limitant aspect that do not provide access to T1 transition / shear decomposition, then openly discuss this limitation, as it would be important for the journal readership to grasp what is doable and what is not doable with such methods.

3. I realise it would also be relevant to show the pattern of cell apical area in all the contexts. This for sure is accessible in the dataset and would probably help to understand the dynamics of would closure.

Other minor point :

© 2024. Published by The Company of Biologists under the terms of the Creative Commons Attribution License (https://creativecommons.org/licenses/by/4.0/).

1. The speed pattern in the WT context (Figure 2,C,F) seems to reveal a gradient of speed from the wound to most distant location (with surprisingly faster movement away from the wound). This gradient of speed should generate some strain and a gradient of cell compaction. Have the authors noticed anything along this line ? (specially related to point 3 above, that should be visible in the pattern of cell area).

2. Right now it is unclear what is represented on the heat maps: the median or the average of the different movies ? It would be good to provide this information in the legends.

3. It would also be interesting to provide some ideas on the variability of the wound healing process from tissue to tissue. One way could be to represent the standard deviation heatmap for all the features, but that could also be hard to read. Alternatively, could the authors provide in one supplementary figure the features heat map for each single movie at least for the WT large wounds ?
That would help to appreciate the level of variability between samples and also visualise which elements are on the contrary extremely reproducible.

Reviewer 2

Advance summary and potential significance to field

This manuscript “AI reveals a damage signaling hierarchy that coordinates different cell behaviors driving wound re-epithelialization” by Turley et al., addresses a bottleneck in quantifying in vivo high resolution imaging data and studying complex combinations of cell behaviors that characterize any in vivo response. The tools and visualization approaches the authors use will be a useful contribution to the field. The intuitive representation of cell divisions, cell shape and cell migration are really helpful to understand integrated wound response of epithelia in space and time.

Comments for the author

- The deep learning algorithms used seem to work well to do the functions they propose in the images provided. Even a brief description of tests performed to measure the accuracy of detection would be very useful to understand the robustness of the method even though the authors have described this in their earlier publication.

- To understand and differentiate the changes in cell shape and proliferation in response to the wound and in the context of the different genetic perturbations used, it is important to understand the distribution of proliferation and cell shapes (in reference to a random region similar to the wound sizes) in wild-type tissues before wounding. Related to this point, the authors refer to comparisons to unwounded tissue average. For example in the text the authors say “cells rapidly reverted back to a rounder shape such that by 25 mins post wounding the mean cell shape had returned to the unwounded tissue average” but the Fig. 2G referenced here only show tissue after wounding. What does the unwounded tissue average look like? Is the slight pink in Graphs 2D and 2G towards the end time point significant and showing an overall orientation of the cells in the tissue?

Similarly, how often and how dense are the cell divisions in unwounded wild-type pupal wing?

- Perturbations in Trpm, JNK signalling and hemocyte ablation will likely affect homeostatic proliferation rates and cell shapes. Understanding what change in behavior is specifically wound induced is important to make conclusions about causality. Are the lesser events of proliferation in all 3 mutants indicative of a general suppression of proliferation and not specifically related to the wound response? Comparing the frequency and distribution of proliferation and cell shapes in unwounded tissue between control and mutant conditions will help underpin the effects of these signals, specifically in the wound response.

- Since the manuscript aims to describe the relationship and causality of the 3 cell behaviors in the context of the wound response, it would be helpful to perturb these behaviors independent of wound signals and observe the effects on wound closure. Eg: Slowing down or speeding up cell

© 2024. Published by The Company of Biologists under the terms of the Creative Commons Attribution License (https://creativecommons.org/licenses/by/4.0/).

divisions using cell cycle mutants and observing the corresponding effects on the suppression and spatio-temporal burst of proliferations after wounding would greatly advance our understanding of the contributions of specific cell behaviors in a spatio-temporal context.

Minor:

1. In the Movies S1 and Fig. 2B-B'', the E-Cadherin signal in some cells bisects the nuclear signal and does not exactly correspond as they should. This could be because z-projections are shown. While quantifying cell divisions and cell shape from z-projections, how is this problem resolved and each nucleus and corresponding correct cell boundary identified?

2. The figure callout for some panels in Fig.5 is labelled wrong in the text.

Reviewer 3

Advance summary and potential significance to field

The paper by Turley and company entitled "AI reveals a damage signaling hierarchy that coordinates different cell behaviors driving wound re-epithelialization" investigates wound healing in the Drosophila wing epithelium using emerging AI techniques.

Leveraging previous NN methods for segmentation, of cell shapes, and behaviors, the authors perform a quantitative study of cell shape changes, cell divisions, and cell migration during wound healing. Two wound sizes in stereotyped positions are analyzed with respect to distinct genetic perturbations: Calcium inhibition, JNK signaling, and wound inflammatory response. A complex relationship between these signaling pathways and cell behaviors characterized here is presented.

Comments for the author

The work is of interest to a broad audience. I have a few suggestions to the authors below.

Major:

This is a nice quantitative study, and it would extend its audience by better connecting to existing wound healing work in culture. In particular the genetic component added through this study would bring added value to this discussion. Wound healing has been investigated from a quantitative point of view in cell culture (Shvartsman lab, Hufnagel group, W. James Nelson lab, and more recent Gardell lab - I would be happy to provide references if the authors like). All these studies found quantitative evidence for a mechanical link with cell proliferation. The authors might find similar effects here, and could easily add quantifications of mechanical observables such as cell density to their study (I assume this data is already available thanks to the superb segmentation tools).

I'm wondering why the quantitative analysis was often normalized to the maximum. One would expect such a normalization is prone to outliers. Would it be possible to normalize to the 5th percentile of the distribution?

Similarly the averaged q-tensor normalization may be chosen to reflect deviation with respect to round shape, rather than the maximum elongation. This may be achieved by measuring the magnitude of the traceless part vs the magnitude of the whole tensor.

Minor: On page ten, the panel references to figure 5 may be scrambled.

Sebastian Streichan

First revision

© 2024. Published by The Company of Biologists under the terms of the Creative Commons Attribution License (https://creativecommons.org/licenses/by/4.0/).

Author response to reviewers' comments

Reviewer 1 Advance summary and potential significance to field In this study, Turley and colleagues applied various deep-learning based methodologies to perform a systematic and quantitative phenomenology of wound healing in the fly pupal wing. Using tools described in a previous publication, including U-net based improved segmentation and automatic detection of cell division, they performed an exhaustive characterisation in space and time of key drivers of wound healing (cell shape, cell migration, cell division) in a quite large number of movies of healing pupal wing in WT, upon perturbation of calcium signaling, JNK signaling and elimination of haemocytes. While the manuscript may not provide clear mechanistic/molecular understanding of which cellular effectors are affected by these pathways, it is a very interesting proof of concept of the usage of automatise methodology to performed a very thorough and detailed characterisation of phenotype, including subtle differences in term of spatio-temporal distribution of cell behaviour, which to my knowledge was probably never performed to this extend in the context of wound healing.

Thank you; we agree; we think this is a novel way of studying these cell:cell interactions in the context of a healing wound and is a first step in quantifying how each of these signals contribute to the various cell processes that re-epithelialise a wound. We will address mechanistic models (which will require a number of assumptions) in our future studies.

The main novel features is the clear delineation of the effet of JNK signaling of cell shape and cell division (without strong impact on cell movement), the effect of haemocyte on cell migration, and the general impact of calcium on all the process. Despite the absence of detailed mechanisms explaining these quantitative phenotypes, I believe this article could be well positioned for Developmental readership and it exemplifies and sets interesting new standard for quantitative charaterisation of phenotype using AI. Somehow, this was already done in the context of morphogenesis through systematic segmentation and shear decomposition in the context of tissue elongation in the pupal wing, notum and embryo, but I believe this manuscript shows that one can start to extend these exhaustive approaches to a larger range of phenotype and higher number of movies when combined with AI. My main critic at this point is that some of the extracted features are relative hard to interpret in term of “phenotype” since they are themself emerging for a large range of cellular behaviours and I am wondering if other metrics (provided they are accessible with the dataset) may not help to go even deeper in the understanding of the phenotype. I realise that some of the these parameters may not be accessible without very intensive work (specially if these parameters would require flawless segmentation and tracking) and touch then the limit what one can currently do with reasonable manual curation and deep learning (and as such should be openly discussed here, see my suggestions below).

We thank the referee and agree that including other metrics will aid in the interpretation of the data. In fact, we think that this kind of work is only the first step in extracting meaning from these enormously large data sets that will increasingly become part of modern biological research. There are limitations in what we can currently measure, but we have some ideas of how to explore the exciting vista ahead. We have discussed some of these in the resubmitted version – see response to points below. Finally, to put this work in context, we have also added a reference to some of the earlier literature on quantification of morphogenesis in fly embryo and pupae.

Reviewer 1 Comments for the author
Main suggestions:
1.So far, the manuscript has been mostly documenting cell division, cell speed, and cell shape. While each of these features are very relevant, I believe cell speed is relatively hard to interpret as it could emerge from many features, for instance the purse-string tension at the wound that could pull cells, the active crawling of cells (specially at the leading edge of the wound) and / or the movements driven by global cell shape changes and/or T1 transition/cell intercalation. As a result, it is relatively hard to interpret what are the key cellular effectors affected by the different perturbations. To be really exhaustive, one should ideally perform the “shear” decomposition in radial coordinates around the wound in all the context described (which would give a “unambiguous” evaluation of the contribution of different cell behaviour to the closure), but admittedly this would be very tedious in all these movies and cellular contexts as it would require

© 2024. Published by The Company of Biologists under the terms of the Creative Commons Attribution License (https://creativecommons.org/licenses/by/4.0/).

flaw-less segmentation for perfect accurate tracking (if I understood well, the authors did not do that, they just analyse cell shape at different point without considering cell identity other time, which can deal properly with ~5% segmentation error ? This was not crystal clear in the method). As such, I would not necessarily recommend to do this analysis (unless the authors do have fully curated segmentation), but at least this limitation should be clearly discussed, especially if the point of the article is to show what can be done using these automatised methodologies.

We agree; our hope was to set the scene with this pioneering approach. We are unable to segment the tissue perfectly with our methods and so could not track cell identity over time. We have made this limitation clearer in the text, pp 12.

2.Along this line, I realise one key feature not accessible right now is tissue fluidity and the rate of T1. Since this was already shown to play an essential role in wound healing it would have been relevant to extract this feature. But this again requires segmentation and tracking (and may not be easily accessible at this stage).

This too would be very interesting and informative as to how tissues reorganise themselves as they repair a wound. Unfortunately, this is inaccessible in our data, as you rightly suggest. Our approach has been to quantify key aspects of wound healing which don't rely on perfect segmentation, as this is currently not feasible using automated techniques. With this in mind, in our future studies, we plan to develop deep learning algorithms that can detect T1 transitions from dynamic videos using a method similar to our division detection algorithm in our recently published eLife paper, which might circumvent the need for perfect segmentation. We have added a note in this regard in the Discussion section, pp 14.

To summarise point 1 and 2, I am suggesting clarifying what is exactly accessible in the dataset, and if the segmentation/tracking is indeed one limitant aspect that do not provide access to T1 transition / shear decomposition, then openly discuss this limitation, as it would be important for the journal readership to grasp what is doable and what is not doable with such methods.

In the discussion on pp 14 we have now made this limitation clear as well as offering a potential solution that we plan for future studies.

3.I realise it would also be relevant to show the pattern of cell apical area in all the contexts. This for sure is accessible in the dataset and would probably help to understand the dynamics of wound closure.

We agree that studying the cell area changes could also offer insights into how wounds heal. We have previously attempted to study this but struggled to find significant changes post-wounding. We have revisited this data following your comments. This time, rather than looking at cell area, we looked at the deviations in cell density (its reciprocal), which gave a clearer signal. We, of course, agree there should be a strain across the tissue, and our new Supplementary Figure 2 (in C-D) shows an increase in cell density, as you suspected. This data is rather noisy as the cell density across this tissue is highly variable, partly due to various morphogenetic episodes occurring, including the formation of veins. In the future, with a system where a laser is directly attached to a confocal (which we don't currently have), live imaging could be performed before, during and after wounding. Such datasets would directly reveal changes in local density in the wounded region, thus removing the issues related to heterogenous tissue structure. We have commented on this limitation in the discussion, pp 14.

Other minor point:

1.The speed pattern in the WT context (Figure 2,C,F) seems to reveal a gradient of speed from the wound to most distant location (with surprisingly faster movement away from the wound). This gradient of speed should generate some strain and a gradient of cell compaction. Have the authors noticed anything along this line? (specially related to point 3 above, that should be visible in the pattern of cell area).

© 2024. Published by The Company of Biologists under the terms of the Creative Commons Attribution License (https://creativecommons.org/licenses/by/4.0/).

Thank you for pointing this out. As we suggest above, there is some indication that there is some strain and a gradient of cell density which suggest directions for future studies. We have now commented on this in the text pp 6.

2.Right now it is unclear what is represented on the heat maps: the median or the average of the different movies? It would be good to provide this information in the legends.

The heat maps are weighted means of cell behaviours measured from each video using the area of the tissue visible to weight this average. We now explain this in the legends for each of these Figures 2-5.

3.It would also be interesting to provide some ideas on the variability of the wound healing process from tissue to tissue. One way could be to represent the standard deviation heatmap for all the features, but that could also be hard to read. Alternatively, could the authors provide in one supplementary figure the features heat map for each single movie at least for the WT large wounds? That would help to appreciate the level of variability between samples and also visualise which elements are on the contrary extremely reproducible.

Thank you for the suggestion. We have now generated a set of signal-to-noise ratio heatmaps for each of the corresponding cell behaviours and conditions, and have added them to Supplementary Figure 2. We hope this gives a clear indication of the strength of the effect and the variation between samples.

***** Reviewer 2 Advance summary and potential significance to field

This manuscript “AI reveals a damage signaling hierarchy that coordinates different cell behaviors driving wound re-epithelialization” by Turley et al., addresses a bottleneck in quantifying in vivo high resolution imaging data and studying complex combinations of cell behaviors that characterize any in vivo response. The tools and visualization approaches the authors use will be a useful contribution to the field. The intuitive representation of cell divisions, cell shape and cell migration are really helpful to understand integrated wound response of epithelia in space and time.

Reviewer 2 Comments for the author

- The deep learning algorithms used seem to work well to do the functions they propose in the images provided. Even a brief description of tests performed to measure the accuracy of detection would be very useful to understand the robustness of the method even though the authors have described this in their earlier publication.

Thank you. We have now added a brief description of the accuracy of the models and how we measured this, pp 3 in the Methods section of the paper.

- To understand and differentiate the changes in cell shape and proliferation in response to the wound and in the context of the different genetic perturbations used, it is important to understand the distribution of proliferation and cell shapes (in reference to a random region similar to the wound sizes) in wild-type tissues before wounding. Related to this point, the authors refer to comparisons to unwounded tissue average. For example, in the text the authors say “cells rapidly reverted back to a rounder shape such that by 25 mins post wounding the mean cell shape had returned to the unwounded tissue average” but the Fig. 2G referenced here only show tissue after wounding. What does the unwounded tissue average look like? Is the slight pink in Graphs 2D and 2G towards the end time point significant and showing an overall orientation of the cells in the tissue? Similarly, how often and how dense are the cell divisions in unwounded wild-type pupal wing?

Thank you for spotting this. We had mistakenly added “unwounded”, where it should read “returned to the tissue average”. After 25 mins the cells close to the wound have, on average, the same shape as the rest of the tissue. We have clarified this in the text pp 6.

© 2024. Published by The Company of Biologists under the terms of the Creative Commons Attribution License (https://creativecommons.org/licenses/by/4.0/).

Yes, you are right; we see a small orientation of cells towards the wound although this is quite a weak (not significant) signal. This is very clear from signal-to-noise ratio heatmaps that we have now added (Supplementary Figure 2B, E) in response to reviewer 1's comments (see above).

We have added a heatmap of cell behaviours around unwounded tissue (around a "virtual wound") to Supplementary Figure 1, and we now explain in more detail in the text, pp 8, and Methods section, pp 21, how we compare this with repairing wound tissue.

- Perturbations in Trpm, JNK signalling and hemocyte ablation will likely affect homeostatic proliferation rates and cell shapes. Understanding what change in behavior is specifically wound induced is important to make conclusions about causality. Are the lesser events of proliferation in all 3 mutants indicative of a general suppression of proliferation and not specifically related to the wound response? Comparing the frequency and distribution of proliferation and cell shapes in unwounded tissue between control and mutant conditions will help underpin the effects of these signals, specifically in the wound response.

This is an important point. To address this concern, we have added Supplementary Figure 3 showing global cell behaviours over time in unwounded mutant tissues; these are largely consistent for velocity and shape changes with those of control tissue. There was some reduction in cell divisions in trpm-RNAi and JNK-DN perturbed tissue, so we have now corrected for this and included details regarding how we did this in the text, pp 8, and Methods section, pp 21. After adjusting for a smaller number of cell divisions in JNK and Ca++ knockdown tissues, we still see a similar post-wound trend but with a larger reduction in cell division suppression at 40-100mins when comparing control and mutant. This correction increases the difference; thank you for pointing this out.

- Since the manuscript aims to describe the relationship and causality of the 3 cell behaviors in the context of the wound response, it would be helpful to perturb these behaviors independent of wound signals and observe the effects on wound closure. Eg: Slowing down or speeding up cell divisions using cell cycle mutants and observing the corresponding effects on the suppression and spatio-temporal burst of proliferations after wounding would greatly advance our understanding of the contributions of specific cell behaviors in a spatio-temporal context.

Absolutely; this is a good question. As we described in the Discussion section of our initial submission (now pp 13-14.) this is one of our next planned steps. We would also like to add granularity to the hemocyte knockdown experiment by knocking down individual growth factor signals in these cells to determine what might be the key signals they are releasing and what wound cell behaviours they regulate.

Minor:
1.In the Movies S1 and Fig. 2B-B'', the E-Cadherin signal in some cells bisects the nuclear signal and does not exactly correspond as they should. This could be because z-projections are shown. While quantifying cell divisions and cell shape from z-projections, how is this problem resolved and each nucleus and corresponding correct cell boundary identified?

In this epithelium, the cells are longer in depth (z) than width (x and y). Nuclei tend to be located at the base of the cell whereas the E-Cadherin signal is located at the apical surface of the cells. This means that sometimes nuclei and boundaries can intersect after projection. We don't link each cell boundary with a nucleus for this reason.

2.The figure callout for some panels in Fig.5 is labelled wrong in the text.

Thank you pointing out these typos which we have now corrected.

***** Reviewer 3 Advance summary and potential significance to field

The paper by Turley and company entitled "AI reveals a damage signaling hierarchy that coordinates different cell behaviors driving wound re-epithelialization" investigates wound healing in the Drosophila wing epithelium using emerging AI techniques.

© 2024. Published by The Company of Biologists under the terms of the Creative Commons Attribution License (https://creativecommons.org/licenses/by/4.0/).

Leveraging previous NN methods for segmentation, of cell shapes, and behaviors, the authors perform a quantitative study of cell shape changes, cell divisions, and cell migration during wound healing. Two wound sizes in stereotyped positions are analyzed with respect to distinct genetic perturbations: Calcium inhibition, JNK signaling, and wound inflammatory response. A complex relationship between these signaling pathways and cell behaviors characterized here is presented.

Reviewer 3 Comments for the author

The work is of interest to a broad audience. I have a few suggestions to the authors below.

Major:
This is a nice quantitative study, and it would extend its audience by better connecting to existing wound healing work in culture. In particular the genetic component added through this study would bring added value to this discussion. Wound healing has been investigated from a quantitative point of view in cell culture (Shvartsman lab, Hufnagel group, W. James Nelson lab, and more recent Gardell lab - I would be happy to provide references if the authors like). All these studies found quantitative evidence for a mechanical link with cell proliferation. The authors might find similar effects here, and could easily add quantifications of mechanical observables such as cell density to their study (I assume this data is already available thanks to the superb segmentation tools).

We thank the reviewer for this comment. In our previous study in eLife, we found that cell divisions were indeed correlated in space-time. While doing this analysis we tried to find a link between cell density and cell proliferation by calculating a spatiotemporal correlation between cell and division density. Surprising, we found no correlation between these properties. As mentioned earlier, in response to reviewer 1, cell density is highly variable in this tissue due to the development of structures in the wing, and so is quite different from homogenous in vitro cell cultures. These variations across the tissue may be masking effects of lower than typical cell density in a region triggering divisions. As mentioned above, we hope to overcome this issue in future studies using a system where a laser is attached directly to the confocal enabling live-imaging to be performed before, during and after wounding. We now flag up this concern and future ways to mitigate it, in our Discussion, pp 14. These datasets could show a change in density of an area of cells, thus removing effects of heterogenous tissue structure.

I’m wondering why the quantitative analysis was often normalized to the maximum. One would expect such a normalization is prone to outliers. Would it be possible to normalize to the 5th percentile of the distribution?

Yes, we agree, this would be wiser, and we have now changed normalisation to the 5th percentile as you suggest (Methods pp 21)

Similarly, the averaged q-tensor normalization may be chosen to reflect deviation with respect to round shape, rather than the maximum elongation. This may be achieved by measuring the magnitude of the traceless part vs the magnitude of the whole tensor.

The q-tensor is a traceless matrix; we make this clearer in the text and methods, pp 5 and pp 19.

Minor: On page ten, the panel references to figure 5 may be scrambled.

Thank you. We have corrected this now.

We hope these comments and our revisions are satisfactory and that you now find our paper ready for publication in Development.

Best regards

Paul M and co-authors

© 2024. Published by The Company of Biologists under the terms of the Creative Commons Attribution License (https://creativecommons.org/licenses/by/4.0/).

Second decision letter

MS ID#: DEVELOP/2024/202943

MS TITLE: AI reveals a damage signalling hierarchy that coordinates different cell behaviours driving wound re-epithelialisation

AUTHORS: Jake Turley, Francesca Robertson, Isaac V. Chenchiah, Tanniemola B. Liverpool, Helen Weavers, and Paul Martin
ARTICLE TYPE: Research Article

I am happy to tell you that your manuscript has been accepted for publication in Development, pending our standard publication integrity checks.

Reviewer 1

Advance summary and potential significance to field

The authors have described an innovative technic based on AI driven segmetentation, automatic cell division detection and cell tracking to characterise the dynamics of cells during epithelial wound healing in vivo in WT and various perturbed conditions. This proof of concept establish a very interesteing new baseline to dissect quantitatively complex tissue behaviour at high throughput in large movies and provides interesting insights about regulation of wound healing.

Comments for the author

The authors have adressed all my concerns and improved the manuscript and clarified what is achievable with their strategy. I am fully supportive for publication at this stage.

There is just one last very minor point (that do not require re-revision) : I could not find in the method how the signal to noise ratio shown in Figure S2 was calculated. It might be good to add a line about this in the method (and my apologies if I missed it).

Reviewer 2

Advance summary and potential significance to field

The authors have completed all the requested experiments. Congratulations on their exciting work.

Comments for the author

No further revisions recommended. the work is ready to be published

Reviewer 3

Advance summary and potential significance to field

Thanks for the revisions. Just a side note: While I agree the q-tensor is traceless, I still wonder if the average q-tensor is traceless. Minor point that should not be in the way of publication. Congratulations on a nice work.

Sebastian Streichan

Comments for the author

none

© 2024. Published by The Company of Biologists under the terms of the Creative Commons Attribution License (https://creativecommons.org/licenses/by/4.0/).
